# Heavy-ion (^56^Fe) irradiation leads to impaired aortic relaxation prior to atherosclerotic plaque formation in ApoE^−/−^ mice

**DOI:** 10.1093/jrr/rrt190

**Published:** 2014-03

**Authors:** C.R. White, T. Yu, K. Gupta, J.H. Kabarowski, Dennis F. Kucik

**Affiliations:** 1 Department of Medicine, University of Alabama at Birmingham, Birmingham, AL 35294, USA; 2 Department of Pathology, University of Alabama at Birmingham, Birmingham, AL 35294, USA; 3 Department of Microbiology, University of Alabama at Birmingham, Birmingham, AL 35294, USA; 4 Veterans Affairs Medical Center, Birmingham, AL, USA

**Keywords:** cardiovascular, atherosclerosis, ^56^Fe, mouse, aorta, heavy ions

## Abstract

Terrestrial radiation exposure is a well-established risk factor for cardiovascular disease. For example, coronary artery disease and stroke are both well-established adverse effects of therapeutic radiation, especially for breast and head-and-neck cancers [ 1]. Similarly, atomic bomb survivors were significantly more likely to die of cardiovascular disease than their countrymen [ 2]. Even radiation technologists, prior to 1950 (when regulations governing shielding and occupational exposure were less rigorous), had an increased risk of clinically significant atherosclerosis [ 3]. Although the character of the radiation in interplanetary space is very different from that encountered on Earth, there is concern that exposure to this cosmic radiation might pose a similar risk for astronauts. Decreased endothelium-dependent vasodilation is thought to predispose humans to the development of structural vascular changes that precede development of atherosclerosis. Therefore, in this study, we examined the effect of ^56^Fe, an important component of cosmic radiation, on vascular relaxation. At the NASA Space Radiation Laboratory at Brookhaven National Laboratory, 10-week-old apoE^−/−^ mice (an age at which there is little atherosclerotic plaque in the descending aorta) were exposed to 2.6 Gy ^56^Fe, a dose in the range that was shown previously to accelerate the development of atherosclerotic plaques in this mouse model at 13 weeks post-exposure [ 4]. The mice were then transferred to the University of Alabama at Birmingham, where they were housed under standard conditions and fed a normal diet. At 4–5 weeks post-irradiation, aortic rings were isolated and endothelial-dependent relaxation in irradiated mice was compared with that of age-matched controls from un-irradiated apoE mice and from un-irradiated wild-type mice of the same C57BL/6 genetic background. Aortic relaxation was not significantly different between un-irradiated apoE^−/−^ mice and un-irradiated wild-type C57BL/6 mice, consistent with previous studies. ^56^Fe radiation, however, resulted in significantly impaired relaxation in response to acetylcholine. This suggests that heavy-ion radiation exposure leads to impairment of normal vascular reactivity prior to the appearance of atherosclerotic plaques, suggesting that it may be a driving force, rather than a consequence, of radiation-induced accelerated atherosclerosis.
